# Supplementary figures and images for: Deviations from Mendelian Inheritance on Bovine X-Chromosome Revealing Recombination, Sex-of-Offspring Effects and Fertility-Related Candidate Genes
Source: Genes (Basel). 2022 Dec 9;13(12):2322. doi: 10.3390/genes13122322 (PMC9778079; doi:10.3390/genes13122322)

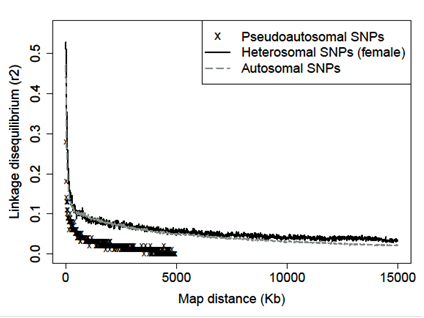

Supplement: Supplementary file 1 [file genes-13-02322-s001.zip › Figure S1.png]
